# Stability of Cantilever Fixed Dental Prostheses on Zirconia Implants

**DOI:** 10.3390/ma15103633

**Published:** 2022-05-19

**Authors:** Nadja Rohr, Reto Nüesch, Rebecca Greune, Gino Mainetti, Sabrina Karlin, Lucia K. Zaugg, Nicola U. Zitzmann

**Affiliations:** 1 Biomaterials and Technology, Department of Research, University Center for Dental Medicine Basel, University of Basel, CH-4058 Basel, Switzerland; rebecca.greune@stud.unibas.ch (R.G.); gino.mainetti@stud.unibas.ch (G.M.); sabrina.maertin@unibas.ch (S.K.); 2 Department of Reconstructive Dentistry, University Center for Dental Medicine Basel, University of Basel, CH-4058 Basel, Switzerland; nueesch.reto@unibas.ch (R.N.); lucia.zaugg@unibas.ch (L.K.Z.); n.zitzmann@unibas.ch (N.U.Z.)

**Keywords:** fracture load, cantilever, FDP, biaxial flexural strength, zirconia implant, chewing simulation

## Abstract

Background: The objective was to determine the optimal connector size and position within zirconia disks for implant-supported cantilever fixed dental prostheses (ICFDP). Methods: Two-unit ICFDPs (*n* = 60) were designed for the premolar region with connector sizes of either 9 or 12 mm^2^ and positioned in the enamel or dentin layer of two different types of zirconia disks. The restorations were milled and cemented onto zirconia implants. After simulated chewing for 1.2 Mio cycles, the fracture load was measured and fractures were analyzed. Results: No fractures of ICFDPs or along the implants were detected after simulated aging. The mean fracture load values were significantly higher for a connector size of 9 mm^2^ (951 N) compared with 12 mm^2^ (638 N). For the zirconia material with a higher biaxial flexural strength, the fracture load values were increased from 751 to 838 N, but more implant fractures occurred. The position within the zirconia disk did not influence the fracture load. Conclusions: A connector size of 9 mm^2^ and a zirconia material with a lower strength should be considered when designing ICFDPS on zirconia implants to reduce the risk of fractures along the intraosseous implant portion.

## 1. Introduction

Cantilever fixed dental prosthesis (FDP) retained at one dental implant are frequently indicated for edentulous spaces with two missing teeth or in free-end situations, due to limited space for two implants, soft or hard tissue deficiencies requiring complex surgical procedures, or financial reasons. The 5-year survival rate of implant-supported cantilever FDPs (ICFDP) varies between 89.9 and 92.7%, with implant fracture being the most common reason for failure [1]. Technical complications of ICFDPs placed in the posterior region have been reported [2,3], with distal cantilevers at higher risk than mesial cantilevers [4].

Metal–ceramic or zirconia (zirconium dioxide) are currently the materials of choice for ICFDPs [5]. Zirconia is a polymorph material that can exist in three different crystal configurations: monoclinic, tetragonal, and cubic [6]. Adding yttria (yttrium oxide) stabilizes the crystal structure of the zirconia (yttria-stabilized zirconia), with the amount of added yttria (mol%) determining the crystal phase and consequently the material strength. Zirconia with a 3 mol% of yttria stabilizes the material in a tetragonal state (3Y-TZP: 3 mol% yttria-stabilized tetragonal zirconia). The material 3Y-TZP is traditionally used as an opaque framework material due to its high strength of around 1200 MPa. Zirconia with 4 or 5 mol% of yttria has a higher cubic content and is more translucent (4Y/5Y-PSZ: partially stabilized zirconia). Due to the pronounced risk for framework fractures and chippings of veneered 3Y-TZP FDPs fixed on implants, monolithic zirconia may be considered [5]. For esthetic reasons, multi-layered 4Y-PSZ or 3Y-TZP/5Y-PSZ disks with translucent layers and color grading have been introduced. However, the strength of translucent zirconia ranges between 400 to 1000 MPa—depending on the product—and may even vary within multi-layered disks [7,8,9].

Dental implants made of titanium or titanium-zirconium alloy are currently the most prevalent, with reported survival rates above 90% after 10 to 15 years [10,11,12,13]. One-piece zirconia implants can be considered a promising alternative to titanium implants with cumulative 5-year implant survival rates between 94 and 98% when restored with single crowns or three-unit FDPs [14,15,16]. When planning ICFDPs made of zirconia on one-piece zirconia implants, data from in vitro investigations are needed, as zirconia is more sensitive to tensile loads than titanium.

The objective of this study was to determine the optimal connector size and position within different zirconia disks for ICFDPs on zirconia implants in the premolar region in vitro, over 5 years of simulated function. The null-hypotheses were that (1) varying connector size does not affect fracture load values, and fracture load is neither influenced by (2) the biaxial flexural strength of the restorative zirconia material, nor (3) by the position of the restoration in the dentin or enamel layer of the zirconia disk.

## 2. Materials and Methods

The replacement of a mesial premolar with an ICFDP on a zirconia implant was simulated in vitro. ICFDPs were manufactured to test the effect of connector size, zirconia material and position within the zirconia disk. Artificial aging replicating 5 years of function was performed with a chewing simulator. Afterwards, fracture load testing was conducted, and fractures were analyzed.

### 2.1. Chewing Simulation

Sixty zirconia implants (Pure, Straumann, Basel, Switzerland) of diameter 4.1 mm and length 12 mm were embedded in an epoxy resin (RenCast CW 20/Ren HY 49, Huntsman Advanced Materials, Duxford, UK) that has a similar elastic modulus to human bone while keeping a 3 mm distance from the nominal bone level of the implant, according to ISO standard 14801:2016.

**Figure 1:**
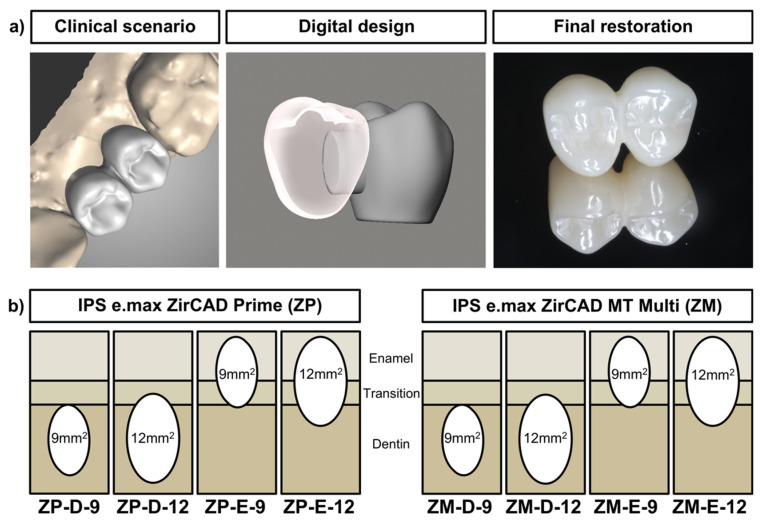
Set-up of the study: (**a**) implant cantilever fixed dental prostheses design; (**b**) specimens were designed with a connector size of either 9 or 12 mm^2^ and positioned in the disc within the enamel (E) or dentin (D) layer of zirconia material ZP and ZM.

FDPs for an implant in the premolar region (tooth 35) with a mesial cantilever (tooth 34) were designed with an oval shaped connector size of either 9 mm^2^ or 12 mm^2^ (Meshmixer V3.5.474, Autodesk, San Rafael, CA, USA). The designs were saved in a StereoLithography (STL) format and transferred to a lab software (Cares Visual2020, Straumann, Basel, Switzerland). Two different zirconia materials were tested; a 3Y-TZP with a translucent 5Y-PSZ enamel layer (ZP; IPS e.max ZirCAD Prime, Ivoclar, Schaan, Liechtenstein) and a multilayered 4Y-PSZ material (ZM; IPS e.max ZirCAD MT Multi, Ivoclar, Schaan, Liechteinstein). The ICFDPs were nested in the zirconia blanks in the lab software, either in the top-most position (enamel layer, E) or bottom (dentin layer, D) position (Figure 1). After milling (ceramill D-series, Amann Girrbach, Pforzheim, Germany), the ICFDPs were carefully removed from the blanks and sintered according to the recommendations of the manufacturer (Zircomat 6100, Vita, Bad Säckingen, Germany). Occlusal surfaces of the specimens were then polished with rotating polishers (HP 9771C 170, HP 9771M 170, HP 9771F 170, Hager & Meisinger, Neuss, Germany). Six ICFDPs were produced per group.

The intaglio surface of the ICFDPs were then air-abraded with alumina particles of 50 µm, cleaned in an ultrasonic bath (TPC-15, Telsonic AG, Bronschhofen, Switzerland) with 70% ethanol for 4 min and dried with oil-free air. After drying, a ceramic primer (Clearfil Ceramic Primer Plus, Kuraray Noritake, Okayama, Japan) containing 10-methacryloyloxydecyl dihydrogen phosphate (MDP) was applied on the abutment surface of the zirconia implants and the intaglio surface of the ICFDPs using a microbrush (Vita Adiva Microbrush, Vita, Bad Säckingen, Germany). ICFDPs were subsequently cemented with an adhesive resin composite cement (Panavia V5, Kuraray Noritake, Okayama, Japan) and placed in a custom-made device applying a load of 2 N for 10 min on each specimen. Excess cement was immediately removed with foam pellets and light-curing of the margins was performed for 60 s (Elipar DeepCure-S, 3M, Neuss, Germany). Specimens were placed in 37 °C water for 24 h directly after cementation.

**Figure 2:**
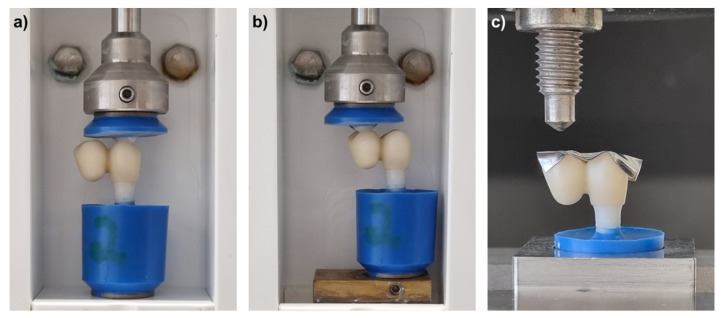
(**a**) Load application during chewing simulation. (**b**) Load application during chewing simulation on cantilever for additional groups ZM-E-9* and ZM-D-9*. (**c**) Load application for fracture load measurements. * = loaded on cantilever during chewing simulation.

All specimens were subjected to a custom-built chewing simulator for 1.2 Mio cycles with a frequency of 1.5 Hz and a load of 49 N to simulate aging by 5 years [17,18]. Simultaneously, a total of 6000 thermal cycles with water of 5 and 55 °C were performed. The load was applied on the occlusal surface in the direction of the implant axis with a polished zirconia antagonist ball of 5 mm in diameter, embedded in epoxy resin (Figure 2a). To analyze the effect of eccentric load application on the cantilever, six additional specimens were produced per group of ZM-E-9* and ZM-D-9*, which were exposed to equal chewing simulation on the occlusal surface of the cantilever instead of the implant crown (Figure 2b). These groups were selected as the least implant fractures were expected due to the lowest connector size and material strength, allowing the focus to be put on the effect on the reconstruction. Afterwards, specimens were inspected under a light microscope (Wild M7A, Leica, Heerbrugg, Switzerland) for fractures.

### 2.2. Fracture Load

Specimens were subjected to fracture load testing directly after chewing simulation and inspection. Using a universal testing machine (Z020, Zwick/Roell, Ulm, Germany), load was applied for all specimens on the occlusal surface of the cantilever in an axial direction (Figure 2c) with a crosshead speed of 1 mm/s until fracture occurred.

Fractured specimens were examined using a 3D scanning laser microscope (VK-X1050, Keyence, Osaka, Japan) and a light microscope (Wild M7A, Leica Heerbrugg, Switzerland). Fractures were classified as Type 1: failure within the ICFDP, or Type 2: failure of the implant. The fracture origin was analyzed with scanning electron microscopy (SEM; ESEM XL-30, Philips, Eindhoven, The Netherlands) at 15 kV for selected fractures using secondary electron (SE) and backscattered electron (BSE) modes.

### 2.3. Biaxial Flexural Strength

To test the influence of the position within the zirconia blanks of the specimens, biaxial flexural strength of the two zirconia materials (ZP and ZM) was measured. Disks with a diameter of 12.5 mm and a height of 1.8 mm were designed (Meshmixer V3.5.474, Autodesk, San Rafael, CA, USA), and nested in either the top enamel layer (E), the middle (M), or the dentin layer at the bottom (D) of the respective zirconia blank (ZP or ZM). Twelve disks were produced for each of the six groups and sintered according to the recommendations of the manufacturer (Zircomat 6100, Vita, Bad Säckingen, Germany). Load-to-fracture of the specimens was obtained according to ISO standard 6872:2015 with the specimen’s surface pointing towards the enamel layer placed downwards on the three supporting balls. A universal testing machine (Z020, Zwick/Roell, Ulm, Germany) with a crosshead speed of 1 mm/min was used and biaxial flexural strength was calculated. Afterwards, two specimens per group were selected and thermally edged for 1 h at 1250 °C (Zircomat 6100, Vita, Bad Säckingen, Germany) to visualize the grain structure. Specimens were gold-sputtered and images of the surfaces facing towards the enamel layer were obtained with SEM (ESEM XL-30, Philips, Eindhoven, The Netherlands). Elemental composition (Al, Hf, O, Y, Zr) of the surfaces was obtained using energy-dispersive X-ray spectroscopy (EDX; Genesis, EDAX, Mahwah, NJ, USA).

### 2.4. Statistics

Sample size for fracture load testing was chosen based on the outcome of a previous study [19] considering a power of 0.8 and a level of significance of 0.05. This resulted in a minimum sample size of 5 samples per group. As there were 12 places available in the chewing simulator, groups of 6 were used. Mean and standard deviation values were calculated, and normal distribution was checked using Shapiro–Wilk test. For the fracture load values, three-way ANOVA was applied for the factors: connector size, zirconia material, and position. Additional groups loaded on the cantilever to analyze the effect of eccentric load application with ZM-E/D-9 were compared separately with two-way ANOVA. Each ANOVA was followed by Bonferroni post hoc test. For biaxial flexural strength data, two-way ANOVAs were performed to test the effects of zirconia material and position within the blank. Biaxial flexural strength was additionally analyzed using Weibull statistics. The level of significance was set to 0.05 (StatPlus Pro V6.1.25, AnalystSoft, Alexandria, VA, USA).

## 3. Results

### 3.1. Fracture Load and Fracture Analysis

**Figure 3:**
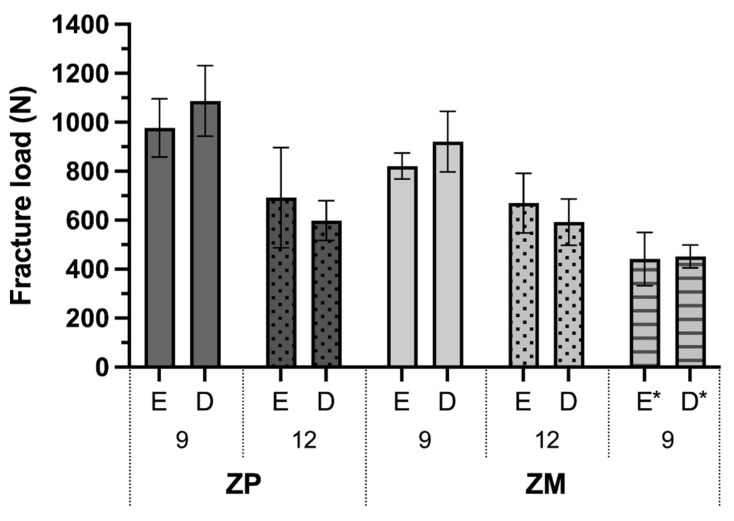
Fracture load mean and standard deviations of ICFDPs on zirconia implants. ICFDPs were made of either 3Y-TZP/5Y-PSZ (ZP) or 4Y-PSZ (ZM) with connector sizes of 9 or 12 mm^2^ positioned in the enamel (E) or dentin (D) layer of the disks. * = loaded on cantilever during chewing simulation.

All specimens survived the chewing simulation without detectable fractures to the ICFDP or the dental implant under the light microscope. Fracture load means and standard deviations are presented in Table 1 and Figure 3. Overall, the fracture load values were significantly affected by the connector size (*p* < 0.001) and zirconia material (*p* = 0.02), but not the position within the zirconia disk (*p* = 0.797). The fracture load values with material ZP were significantly higher (838 ± 245 N; *p* = 0.020) than for ZM (751 ± 167 N). Implant fractures (Type 2) occurred in 83% of the ZP specimens and 29% of the ZM specimens. The fracture load values were significantly higher with 9 mm^2^ connectors (951 ± 145 N) than with 12 mm^2^ connectors (638 ± 133 N; *p* < 0.001). Furthermore, fewer implant fractures occurred with 9 mm^2^ connectors (38%, only ZP) than with 12 mm^2^ connectors (75%, ZP and ZM, Table 1). Type 1 fractures were found at an overall mean fracture load value of 771 ± 238 N and Type 2 at 733 ± 231 N.

Loading the cantilever during the chewing simulation (specimens ZM-E-9* and ZM-D-9*) significantly decreased the mean fracture load (446 ± 81 N) compared with equivalent samples ZM-E-9 and ZM-D-9 (871 ± 105 N) loaded on the implant crown (*p* < 0.001). While implant fractures were not observed in ZM-E-9 and ZM-D-9 specimens, 50% of the specimens that underwent loading of the cantilever had fractures of the implant. The position within the disk had no significant effect (*p* = 0.145).

**Figure 4:**
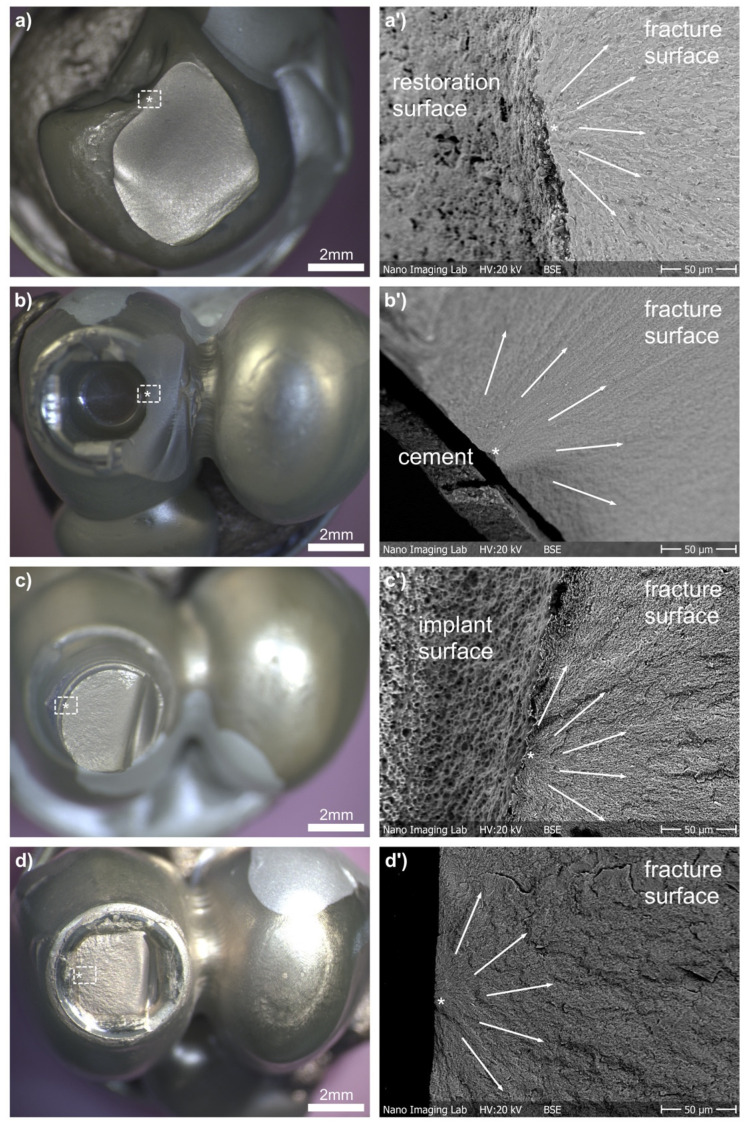
Analysis of fracture origin (*) in light microscopy and scanning electron microscopy (’) images for the different failures of Type 1 (**a**) connector, and (**b**) crown margin; and Type 2 (**c**) implant embedding height, and (**d**) implant abutment height.

Most failures occurring in the ICFDPs started from the cervical part where the ICFDPs were cemented onto the implant abutments and facing the side of the connector (Figure 4a). Only two ICFDPs of group ZM-E-9 failed by complete separation along the connector (Figure 4b), where the fracture origin started from the occlusal side in the zone of the largest tension.

Implant failure (Type 2) occurred mainly at the level of the embedding material, 3 mm below the nominal bone level. A typical compression curl was visible on the failure site of all fractured implants (Figure 4c). Three implants fractured at the abutment level displaying similar fracture patterns (Figure 4d).

### 3.2. Biaxial Flexural Strength

**Figure 5:**
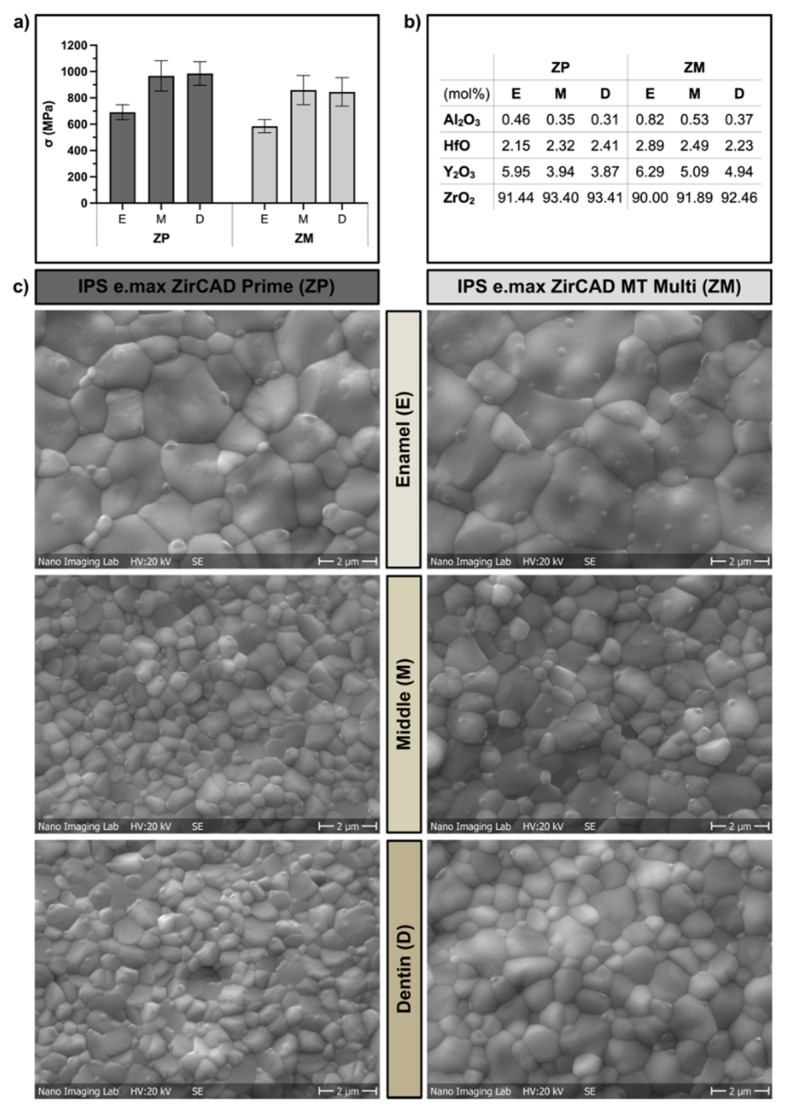
(**a**) Biaxial flexural strength mean values and standard deviations for the different layers (E: enamel, M: middle, D: dentin) of the zirconia disks of materials ZP and PM; (**b**) EDX analysis of oxides present on the surface of specimens of ZP and ZM in mol%; (**c**) scanning electron microscopy of thermally etched zirconia surfaces of biaxial flexural strength specimens of zirconia materials ZP and ZM obtained from the enamel (E), middle (M) and dentin (D) layers of the disks. Grain sizes > 1 µm is an indicator for the presence of cubic crystal phase.

Biaxial flexural strength values were significantly affected by the zirconia material (*p* < 0.001) and the position within the disk (*p* < 0.001). The mean values and standard deviations are presented in Figure 5a. Comparing groups of the same position (E, M, D), the flexural strength values for material ZP (E: 691 ± 57 MPa, M: 969 ± 103 MPa, D: 982 ± 91 MPa) were significantly higher than for ZM (E: 585 ± 49 MPa, M: 842 ± 86 MPa, D: 846 ± 108 MPa; *p* < 0.001). Positioning of the specimens within the enamel layer E of the disk resulted in significantly lower biaxial flexural strength values compared with group M (*p* < 0.001) and D (*p* < 0.001), while no significant difference was found between group M and D (*p* = 1.0). The calculated Weibull characteristic strength was as follows: ZP (E: 717 MPa, M: 1019 MPa, D: 1025 MPa) and ZM (E: 606 MPa, M: 879 MPa, D: 892 MPa). The Weibull characteristic strength was overall around 5% higher than the mean biaxial flexural strength.

Scanning electron microscopy images of the thermally etched samples are shown in Figure 5c. Grains larger than 1 µm are clearly visible for ZP-E and ZM-E and indicate the presence of the cubic phase. The respective elemental composition found using EDX was calculated in oxide content and is displayed in Figure 5b. The content of yttria within the zirconia materials was highest within the enamel layer (6 mol%) for both ZP and ZM. The yttria content decreased to 4 mol% for ZP and 5 mol% for ZM in the dentin layer. Other oxides found were alumina and hafnium oxide.

## 4. Discussion

The objective of this study was to determine the optimal connector size, restorative material, and position within the zirconia disk for ICFDP of either 4Y-PSZ or 3Y-TZP/5Y-PSZ on zirconia implants in the premolar region in vitro over 5 years of simulated function. It was found that the highest fracture load values (870 N) for an ICFDP with the lowest risk for implant fractures after simulated aging can be expected with a 9 mm^2^ connector size milled out of a 4Y-PSZ material. The first hypothesis that fracture load is not affected by connector size was rejected, as higher fracture load values were obtained with 9 mm^2^ connectors compared with 12 mm^2^ connectors. The second hypothesis that fracture load is not influenced by the biaxial flexural strength of the restorative zirconia material was also rejected, as the 3Y-TZP/5Y-PSZ (ZP) material displayed a higher biaxial flexural strength with higher fracture load values compared with the 4Y-PSZ (ZM) material. The positioning of the ICFDP within the zirconia disks did not, however, significantly affect the fracture load values, and the third null-hypothesis was confirmed.

Surprisingly, the larger connector size of the FDPs decreased the fracture load of the samples significantly and increased the risk of implant fractures. A possible explanation is that in ICFDPs with 12 mm^2^ connectors, the stress build-up with load peaks arose at the pivot point where the implant was embedded, as no buffering was possible within the stiff connector during fracture load application on the cantilever. In most cases, fractures within the ICFDPs started from the implant crown margin and progressed towards the connector. This is the area where the highest compressive strength accumulated. For zirconia single crowns on titanium implants it has been observed that restorations with thin walls at crown margins were especially prone to such contact fractures [20]. In the current study, only two ICFDPs of group ZM-E-9 fractured through the connector, initiated from the point of highest tension. The connector itself is not, therefore, the most probable fracture spot, but its size plays a crucial role in how stress is absorbed and transmitted along the restoration and to the implant.

For three-unit FDPs, manufacturers recommend connector sizes of 9 mm^2^ for ZP and 16 mm^2^ for ZM. Although 9 and 12 mm^2^ were planned within the software, the actual connector size may have been slightly increased as a result of the machine’s calculated milling path and limited access of the drills to narrow spaces in the connector area.

The overall fracture load values of the ICFDPs with the 3Y-TZP/5Y-PSZ material (ZP) were significantly higher (838 MPa vs. 751 MPa with the 4Y-PSZ (ZM) material); however, implant fractures occurred with most of the ZP specimens (83% vs. 29% of ZM specimens). Hence, to avoid implant fractures with the consequence of challenging implant removal surgery, a restorative material for the ICFDP with a lower strength should be chosen. Cantilever restorations on zirconia implants are currently not approved by the implant manufacturers. For titanium implants, cantilever restorations are a common treatment option with 5-year-survival rates of between 89.9% and 92.7%. However, implant fractures are reported as the main cause for failure [1]. When restoring a titanium implant with a cantilever, the implant-fracture probability increases with a factor of 2.5 compared to single crowns [21]. For clinically failed zirconia implants that were restored with a single implant crown, fracture analysis revealed a similar fracture behavior with compressive curl formation as found in the present study [22]. This failure type is a typical phenomenon of flexural/bending fractures associated with strong occlusal contacts beyond the implant axis as simulated here. Zirconia is sensitive to subcritical crack growth, meaning that small defects propagate continuously at a low rate even when submitted to a stress intensity factor below the fracture toughness threshold (K_1c_), which subsequently leads to failure of the implants [23].

In the present study, the positioning of the ICFDPs within zirconia disks did not affect the fracture load significantly for either zirconia materials, while the biaxial flexural strength of the specimens in the translucent enamel layer was lower than in the middle or dentin layer. For ZP, the manufacturer discloses a lower biaxial flexural strength of 650 MPa in the top 3 mm of the 5Y-PSZ enamel layer compared with the 3Y-TZP dentin layer (1200 MPa) that is attributed to the higher yttria content and consequently higher cubic phase of the enamel layer. These specifications are consistent with the SEM and EDX findings of the present study; however, the biaxial flexural strength of the dentin layer was lower (976 MPa) in the present study. The manufacturer characterizes ZM as 4Y-PSZ material with a biaxial flexural strength of 650 MPa in the enamel and 850 MPa in the dentin layer. The obtained biaxial flexural strength reported here was slightly lower (585/844 MPa). The SEM analysis revealed the presence of grains > 1 µm that occur with the cubic phase in the enamel layer. EDX analysis showed that the yttria content within all layers was higher (ZP 4–6 mol%, ZM 5–6 mol%) than the information given by the manufacturer (ZP 3/5 mol%, ZM 4 mol%). It is possible, however, that thermal etching of the samples may have enhanced the presence of yttria on the surfaces as crystal phase transformations can occur at the applied temperature of 1250 °C [24].

A previous study confirmed lower flexural strength of multicolored zirconia disks for the translucent enamel layer, while even lower values were found within the transition layer [9]. Manufacturers are encouraged to transparently communicate the actual layer compositions, grain sizes, and strengths, as those specifications are crucial for technicians to correctly choose the material for the respective indications. Further, choosing a zirconia material has become challenging lately due to new developments of multilayered disks and available classification systems based on zirconia generation, yttrium content (mol% versus weight%), or ISO standard EN 6872:2015.

In the present study, all implants survived the chewing simulation which is reported to simulate a clinical function of 5 years [17,18]. Previous data reported similar fracture loads before and after chewing simulation for 2.5 Mio cycles with 98 N for 4-unit FDPs of 3Y-TZP, 4Y-PSZ and 5Y-PSZ materials [25]. As the objective of this study was to test the effect of varying the parameters in the ICFDP design, the load during chewing simulation was applied in axial implant direction on the abutment crown. Two groups were aged by load application on the cantilever to increase the stress on the implant, which decreased the fracture load from 871 to 446 N. Although the implant’s strength was reduced by almost 50% by non-axial loading, the strength is sufficient to resist expected normal chewing forces of 110 to 125 N [26] with maximum peaks of 300–1000 N in the posterior area [27,28]. To limit implant weakening, occlusal contact on the cantilever should be avoided. Although ICFDPs are not cleared for the use on one-piece zirconia implants by the manufacturers, they may be considered for certain limited indications in the future when restored with a lower strength zirconia material.

Despite the in vitro character of the study, relevant and surprising information was obtained on how the material and design of the ICFDP affect the fracture load, as these parameters are not legitimate to be simulated in a clinical trial. A finite element analysis would provide further information on critical loads and predict fracture initiation areas. As zirconia implants are also available as two-piece systems, these may be tested in further studies.

## 5. Conclusions

For cantilever FDPs of zirconia with varying connector size, zirconia material and position within the disk cemented on one-piece zirconia implants, it can be concluded that:

- Higher fracture load values are obtained for cantilever restorations with a 9 mm^2^ than for a 12 mm^2^ connector.
- Fracture load values are significantly higher for ICFDPs made of 3Y-TZP/5Y-PSZ than for 4Y-PSZ disks, while implant fractures are also increased with high-strength zirconia restorations.
- The position of the ICFDP within the enamel or dentin layer of the zirconia disk does not affect fracture load values significantly.

## Figures and Tables

**Table 1:** Fracture load values of each specimen and mean and standard deviations after chewing simulation. Fracture occurring to each specimen are marked with Type 1 (FDP, light grey) or Type 2 (implant, dark grey).

Material	ZP				ZM					
Connector size (mm^2^)	9		12		9		12		9	
Disk position	E	D	E	D	E	D	E	D	E *	D *
										
**1**	744	981	1036	480	754	957	513	617	320	397
**2**	1008	997	584	576	772	927	723	759	331	388
**3**	1087	1303	639	561	820	1058	804	599	601	479
**4**	1005	927	839	606	882	784	525	509	523	460
**5**	1020	1186	550	717	881	1037	751	500	452	494
**6**	1000	1127	504	648	816	766	706	565	419	492
**mean**	977	1087	692	598	821	921	670	592	441	452
**sd**	119	144	205	81	53	124	122	95	109	47
										
**Fracture types**	Type 1	Type 2								

E: enamel, D: dentin, ZP: 3Y-TZP/5Y-PSZ, ZM: 4Y-PSZ; * = loaded on cantilever during chewing simulation.

## Data Availability

The data presented in this study are available on request from the corresponding author.

## Abbreviations

3Y-TZP	3 mol% yttria-stabilized tetragonal zirconia
4Y/5Y-PSZ	4/5 mol% yttria partially stabilized zirconia
Al	aluminum
BSE	backscattered electron
D	dentin layer
E	enamel layer
EDX	energy-dispersive X-ray spectroscopy
FDP	fixed dental prosthesis
HF	hafnium
ICFDP	implant-supported cantilever fixed dental prostheses
M	middle layer
MDP	10-methacryloyloxydecyl dihydrogen phosphate
O	oxygen
SE	secondary electron
SEM	scanning electron microscopy
Y	yttrium
ZM	IPS e.max ZirCAD MT Multi group
ZP	IPS e.max ZirCAD Prime group
Zr	zirconium
